# Double Trouble: New-Onset Graves’ Disease Presenting Twice with Thyroid Storm in the Setting of Acute Abdomen

**DOI:** 10.51894/001c.166021

**Published:** 2026-07-31

**Authors:** Ashutosh Suresh

**Affiliations:** 1 Internal Medicine, Garden City Hospital

## 125

### INTRODUCTION

Thyroid storm is a rare but life-threatening complication of thyrotoxicosis, often triggered by infections, surgery, or physiological stressors. This report describes a case of new-onset Graves’ disease in which thyroid storm was triggered twice—first by acute appendicitis and later by a psoas abscess.

### BACKGROUND

A 41-year-old man with a family history of hyperthyroidism, not on any medications, presented with dyspnea, fever, abdominal pain, diarrhea, palpitations, and delirium for two days. His vital signs showed a heart rate of 170 bpm, blood pressure of 122/76 mmHg, respiratory rate of 40 breaths/min, and a temperature of 98.9°F. An EKG revealed atrial fibrillation, controlled with IV diltiazem. Labs showed leukocytosis (18.4 × 10⁹/L), thrombocytopenia (97 × 10⁹/L), total bilirubin of 1.6 mg/dL, and ALP of 128 U/L. Thyroid function tests confirmed thyrotoxicosis with suppressed TSH (0.009 µIU/mL), elevated total T3 (240 ng/dL), free T3 (4.2 pg/mL), and free T4 (3.7 ng/dL), alongside elevated thyroid autoantibodies, confirming Graves’ disease. A Burch-Wartofsky score of 75 confirmed thyroid storm. Imaging revealed acute appendicitis with perforation and a pericecal abscess. He was treated with IV fluids, propylthiouracil, corticosteroids, lopressor, ceftriaxone, and metronidazole, followed by laparoscopic appendectomy with JP drain placement.

Ten days post-discharge, despite medication compliance, he returned with fever (104.4°F), chills, and abdominal pain. His heart rate was 130 bpm, respiratory rate 20 breaths/min, and blood pressure 122/72 mmHg, with a Burch-Wartofsky score of 95. Labs showed TSH <0.008 µIU/mL, free T4 of 2.7 ng/dL, and WBC of 19 × 10⁹/L. Imaging revealed a large right psoas abscess, which was drained percutaneously. He was treated with IV antibiotics, methimazole, propranolol, and corticosteroids. Following clinical improvement, he was discharged with medications and outpatient follow-up.

### DISCUSSION

This case is unique as the patient experienced thyroid storm twice due to separate intra-abdominal infections. While thyroid storm itself can mimic acute abdomen, missing an underlying surgical cause can be fatal. Physicians should maintain a low threshold for evaluating surgical causes in hyperthyroid patients presenting with acute abdomen. This highlights the importance of early recognition and treatment of thyroid storm, its precipitants, and coexistent autoimmune pathologies to significantly reduce mortality.

